# Associations between *ADIPOQ* polymorphisms and coronary artery disease: a meta-analysis

**DOI:** 10.1186/s12872-019-1041-3

**Published:** 2019-03-18

**Authors:** Xia Zhang, Yan Jun Cao, Hong Yu Zhang, Hongliang Cong, Jian Zhang

**Affiliations:** 1 Department of Cardiovascular Medicine, Baodi Clinical College of Tianjin Medical University, Tianjin, China; 2 Department of Cardiovascular Medicine, Tianjin chest Hospital, No. 261 Taierzhuang South Road, Tianjin, 300000 Jinnan District China; 3 Department of Orthopaedics, Baodi Clinical College of Tianjin Medical University, Tianjin, China

**Keywords:** Adiponectin (*ADIPOQ*), Genetic polymorphisms, Coronary artery disease (CAD), Meta-analysis

## Abstract

**Background:**

Whether adiponectin (*ADIPOQ*) polymorphisms are associated with coronary artery disease (CAD) remain controversial. Therefore, we performed this meta-analysis to better explore potential roles of *ADIPOQ* polymorphisms in CAD.

**Methods:**

PubMed, Web of Science, Embase and CNKI were searched for eligible studies. Odds ratios (ORs) and 95% confidence intervals (CIs) were calculated.

**Results:**

Totally 45 studies were included for pooled analyses. A significant association with the susceptibility to CAD was detected for rs2241766 (dominant model: *p* = 0.0009, OR = 0.82, 95%CI 0.73–0.92; recessive model: *p* = 0.04, OR = 1.29, 95%CI 1.02–1.64; allele model: *p* < 0.0001, OR = 0.80, 95%CI 0.73–0.88) polymorphism in overall population. Further subgroup analyses by ethnicity showed that rs1501299 polymorphism was significantly associated with the susceptibility to CAD in East Asians, whereas rs2241766 polymorphism was significantly associated with the susceptibility to CAD in Caucasians, East Asians and South Asians.

**Conclusions:**

Our findings indicated that rs1501299 and rs2241766 polymorphisms both affect the susceptibility to CAD in certain populations.

## Background

Coronary artery disease (CAD) is the leading cause of death and disability worldwide [1, 2]. To date, the exact pathogenesis of CAD remains largely unknown. Nevertheless, plenty of evidences demonstrated that genetic factors are crucial for the development of CAD. First, family clustering of CAD was observed extensively, and past twin studies showed that the heredity grade of CAD was over 50 % [3, 4]. Second, numerous genetic variants were found to be associated with an increased susceptibility to CAD by previous genetic association studies, and screening of common causal variants was also proved to be an efficient way to predict the individual risk of developing CAD [5, 6]. Overall, these findings jointly indicated that genetic predisposition to CAD is important for its occurrence and development.

Adiponectin (ADIPOQ), an adipocytokine that regulates energy and material metabolism, is implicated in the development of multiple metabolic disorders including obesity and type II diabetes. And it was evident that these two common metabolic disorders were associated with an increased risk of CAD [7]. Furthermore, previous studies demonstrated that adipoenctin have both anti-atherogenic and anti-inflammatory property [8, 9]. Moreover, the expression level of adiponectin was also significantly decreased in CAD patients [10, 11]. Overall, these evidences jointly suggested that adipoenctin might exert favorable protection effects against CAD. Therefore, functional *ADIPOQ* genetic polymorphisms, which may alter the expression level of adiponectin, may also affect individual susceptibility to CAD. Recently, some pilot studies already investigated associations of two common functional *ADIPOQ* polymorphisms, rs1501299 and rs2241766, with the susceptibility to CAD. However, the results of these studies were not consistent, especially when they were conducted in different populations [12–19]. Previous studies failed to reach a consensus regarding associations between *ADIPOQ* polymorphisms and CAD partially because of their relatively small sample sizes. Thus, we performed the present meta-analysis to explore the relationship between *ADIPOQ* polymorphisms and CAD in a larger pooled sample size. Additionally, we also aimed to elucidate the potential effects of ethnic background on associations between *ADIPOQ* polymorphisms and CAD.

## Methods

The current meta-analysis followed the Preferred Reporting Items for Systematic Reviews and Meta-analyses (PRISMA) checklist [20–22].

### Literature search and inclusion criteria

The combination of following terms: (adiponectin OR ADIPOQ) AND (polymorphism OR variant OR mutation OR genotype OR allele) AND (coronary heart disease OR coronary artery disease OR angina pectoris OR acute coronary syndrome OR myocardial infarction) was used to searched for potentially eligible articles that were published prior to December 1, 2018 in PubMed, Web of Science, Embase and China National Knowledge Infrastructure (CNKI). We also reviewed the reference lists of all retrieved articles for other potentially eligible studies.

To test the research hypothesis of this meta-analysis, included studies must meet all the following criteria: (1) case-control study on associations between *ADIPOQ* polymorphisms (rs1501299 and rs2241766) and CAD; (2) provide genotypic and/or allelic frequency of investigated polymorphisms; (3) full text in English or Chinese available. Studies were excluded if one of the following criteria was fulfilled: (1) not relevant to *ADIPOQ* polymorphisms and CAD; (2) case reports or case series; (3) abstracts, reviews, comments, letters and conference presentations. In the case of duplicate reports by the same authors, we only included the most recent study.

### Data extraction and quality assessment

We extracted the following information from eligible studies: 1. name of the first author; 2. year of publication; 3. country and ethnicity of participants; 4. sample size; and 5. genotypic distributions of *ADIPOQ* polymorphisms in cases and controls. The probability value (*p* value) of Hardy-Weinberg equilibrium (HWE) was also calculated.

We used the Newcastle-Ottawa scale (NOS) to evaluate the quality of eligible studies [23]. The NOS has a score range of zero to nine, and studies with a score of more than seven were thought to be of high quality.

Two reviewers conducted data extraction and quality assessment independently (Xia Zhang and YanJun Cao). When necessary, we wrote to the corresponding authors for extra information. Any disagreement between two reviewers was solved by discussion until a consensus was reached.

### Statistical analyses

In the current study, Review Manager Version 5.3.3 was used to perform statistical analyses. We calculated ORs and 95% CIs to estimate potential associations between *ADIPOQ* polymorphisms and CAD in all possible genetic models, and a *p* value of 0.05 or less was defined as statistically significant. Between-study heterogeneities were evaluated by I^2^ statistic. Random-effect models (REMs) would be used for analyses if I^2^ was greater than 50%. Otherwise, analyses would be performed with fixed-effect models (FEMs). Subgroup analyses by ethnicity and type of disease were subsequently carried out. Stabilities of synthetic results were tested in sensitivity analyses. Publication biases were assessed by funnel plots.

## Results

### Characteristics of included studies

**Fig. 1:**
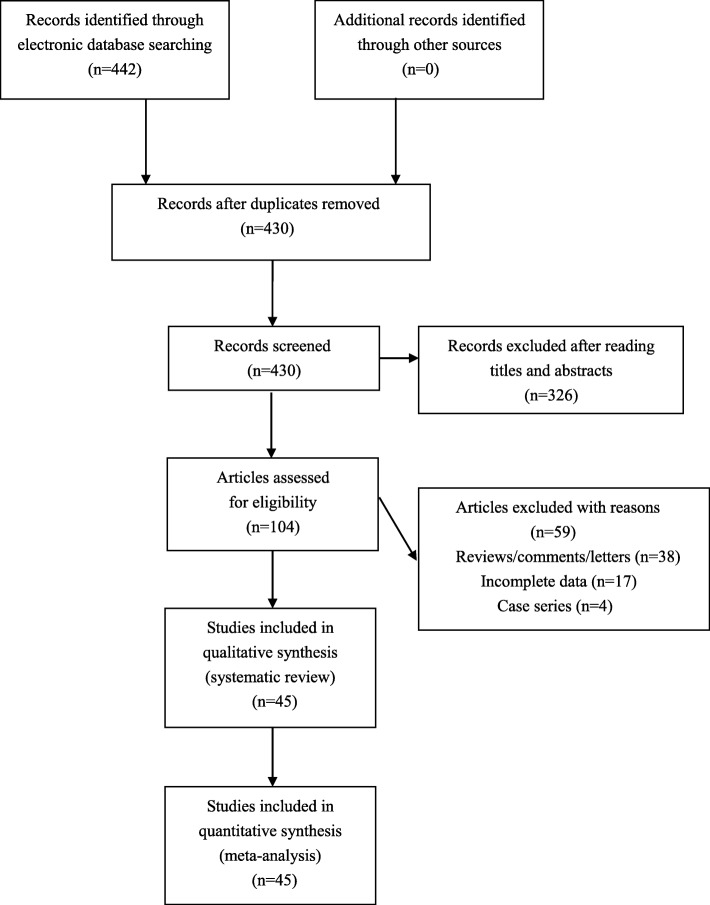
Flowchart of study selection for the present study

We found 442 potential relevant articles. Among these articles, totally 45 eligible studies were finally included for pooled analyses (see Fig. 1). Baseline characteristics of included studies were shown in Table 1.

**Table 1:** The characteristics of included studies

First author, year	Country	Ethnicity	Type of disease	Sample size	Genotype distribution		*P*-value for HWE	NOS score
					Cases Controls			
rs1501299 G/T					GG/GT/TT			
Al-Daghri 2011	Saudi Arabia	South Asian	CAD	123/297	47/57/19	111/142/44	0.897	7
Ambroziak 2018	Poland	Caucasian	MI	188/153	88/72/28	84/59/10	0.933	7
Antonopoulos 2013	Greece	Caucasian	CAD	462/132	220/212/30	66/50/16	0.184	8
Bacci 2004	Italy	Caucasian	CAD	142/234	70/65/7	118/88/28	0.073	7
Boumaiza 2011	Tunisia	Caucasian	CAD	213/108	105/84/23	45/41/18	0.115	8
Chen 2011	China	East Asian	CAD	93/102	54/33/6	61/38/3	0.307	7
Cheung 2014	Hong Kong	East Asian	CAD	182/2010	88/75/19	1103/759/148	0.270	7
Chiodini 2010	Italy	Caucasian	MI	1002/503	530/392/80	239/198/66	0.016	7
De Caterina 2011	Italy	Caucasian	MI	1833/1821	926/746/161	906/767/148	0.419	7
Esteghamati 2012	Iran	South Asian	CAD	114/127	76/30/8	63/47/17	0.095	7
Filippi 2005	Italy	Caucasian	CAD	580/466	287/241/52	266/167/33	0.338	8
Gable 2007	UK	Caucasian	MI	504/557	266/216/22	289/225/43	0.931	8
Ghazouani 2018	Tunisia	Caucasian	CAD	277/269	143/93/41	138/88/43	< 0.001	8
Gui 2012	China	East Asian	CAD	410/431	172/185/53	239/154/38	0.072	8
Hegener 2006	USA	Mixed	MI	341/341	183/134/24	181/143/17	0.093	8
Jung 2006	Korea	East Asian	CAD	88/68	38/43/7	31/32/5	0.399	7
Katakami 2012	Japan	East Asian	MI	213/2424	129/71/13	1229/976/219	0.209	7
Lacquemant 2004	UK	Caucasian	CAD	161/309	82/66/13	169/115/25	0.387	7
Li 2018	China	East Asian	CAD	201/141	67/107/27	64/53/24	0.030	8
Liang 2011	China	East Asian	MI	78/84	30/43/5	48/30/6	0.663	7
Liang 2017	China	East Asian	CAD	960/962	490/388/82	617/300/45	0.275	8
Mohammadzadeh 2016	Iran	South Asian	CAD	100/100	38/55/7	56/42/2	0.063	7
Ohashi 2004	Japan	East Asian	CAD	383/368	185/164/34	190/149/29	0.977	8
Oliveira 2012	Brazil	Mixed	CAD	450/153	209/197/44	62/68/23	0.542	7
Pischon 2007	USA	Mixed	CAD	491/988	266/182/43	485/416/87	0.869	7
Qi 2005	USA	Mixed	CAD	228/594	105/111/12	293/249/52	0.930	7
Rizk 2012	Qatar	South Asian	ACS	142/121	58/64/20	46/59/16	0.667	7
Rodr’ıguez-Rodr’ıguez 2011	Spain	Caucasian	CAD	119/555	69/44/6	287/224/44	0.975	7
Wu 2013	China	East Asian	CAD	188/200	67/108/13	92/90/18	0.545	7
Zhang 2015	China	East Asian	CAD	561/412	309/209/43	214/170/28	0.459	8
Zhang 2018	China	East Asian	CAD	717/612	583/126/8	471/131/10	0.798	8
rs2241766 T/G					TT/TG/GG			
Al-Daghri 2011	Saudi Arabia	South Asian	CAD	122/298	77/35/10	220/72/6	0.969	7
Antonopoulos 2013	Greece	Caucasian	CAD	462/132	359/97/6	99/29/4	0.309	8
Bacci 2004	Italy	Caucasian	CAD	130/220	90/35/5	149/60/11	0.135	7
Boumaiza 2011	Tunisia	Caucasian	CAD	212/104	145/57/10	75/24/5	0.111	8
Chang 2009	Taiwan	East Asian	CAD	600/687	316/238/46	309/399/79	0.606	7
Chen 2011	China	East Asian	CAD	93/102	68/19/6	59/35/8	0.391	7
Cheung 2014	Hong Kong	East Asian	CAD	184/2012	89/83/12	1007/822/183	0.413	7
Chiodini 2010	Italy	Caucasian	MI	1002/503	679/304/19	359/126/18	0.102	7
Di 2011	China	East Asian	CAD	196/124	91/85/20	65/50/9	0.884	7
Du 2016	China	East Asian	CAD	493/304	253/190/50	185/97/22	0.069	8
Esteghamati 2012	Iran	South Asian	CAD	114/127	48/41/25	68/46/13	0.222	7
Foucan 2010	French West Indies	African	CAD	57/159	NA	NA	NA	7
Gable 2007	UK	Caucasian	MI	526/563	360/154/12	384/168/11	0.280	8
Ghazouani 2018	Tunisia	Caucasian	CAD	277/269	181/74/22	182/70/17	0.007	8
Hegener 2006	USA	Mixed	MI	341/341	241/95/5	252/80/9	0.389	8
Jin 2009	China	East Asian	CAD	110/73	53/48/9	50/20/3	0.584	8
Jung 2006	Korea	East Asian	CAD	88/68	41/40/7	34/30/4	0.431	7
Lacquemant 2004	UK	Caucasian	CAD	162/315	109/48/5	249/57/9	0.015	7
Li 2011	China	East Asian	CAD	118/97	51/46/21	54/31/12	0.036	8
Liang 2017	China	East Asian	CAD	960/982	471/382/107	608/308/46	0.387	8
Luo 2010	China	East Asian	CAD	221/100	100/99/22	50/41/9	0.886	7
Mofarrah 2016	Iran	South Asian	CAD	152/72	82/35/35	56/13/3	0.072	8
Mohammadzadeh 2016	Iran	South Asian	CAD	100/100	75/24/1	65/31/4	0.900	7
Nan 2012	China	East Asian	CAD	213/467	115/84/14	237/191/39	0.953	8
Oliveira 2012	Brazil	Mixed	CAD	450/153	323/114/13	117/33/3	0.708	7
Pischon 2007	USA	Mixed	CAD	482/979	374/102/6	759/202/18	0.290	7
Qi 2005	USA	Mixed	CAD	219/599	NA	NA	NA	7
Rizk 2012	Qatar	South Asian	ACS	142/122	62/42/38	56/49/17	0.245	7
Sabouri 2011	Iran	South Asian	CAD	329/241	253/74/2	205/35/1	0.703	7
Xu 2010	China	East Asian	CAD	153/73	78/65/10	50/20/3	0.584	8
Zhang 2011	China	East Asian	CAD	149/167	63/60/26	97/50/20	0.002	7
Zhang 2015	China	East Asian	CAD	561/412	276/235/50	224/164/24	0.399	8
Zhang 2018	China	East Asian	CAD	717/612	500/184/33	456/149/7	0.177	8

Abbreviations: *CAD* Coronary artery disease, *MI* Myocardial infarction, *ACS* Acute coronary syndrome, *HWE* Hardy-Weinberg equilibrium, *NOS* Newcastle-Ottawa scale, *NA* Not available

### Overall and subgroup analyses

**Fig. 2:**
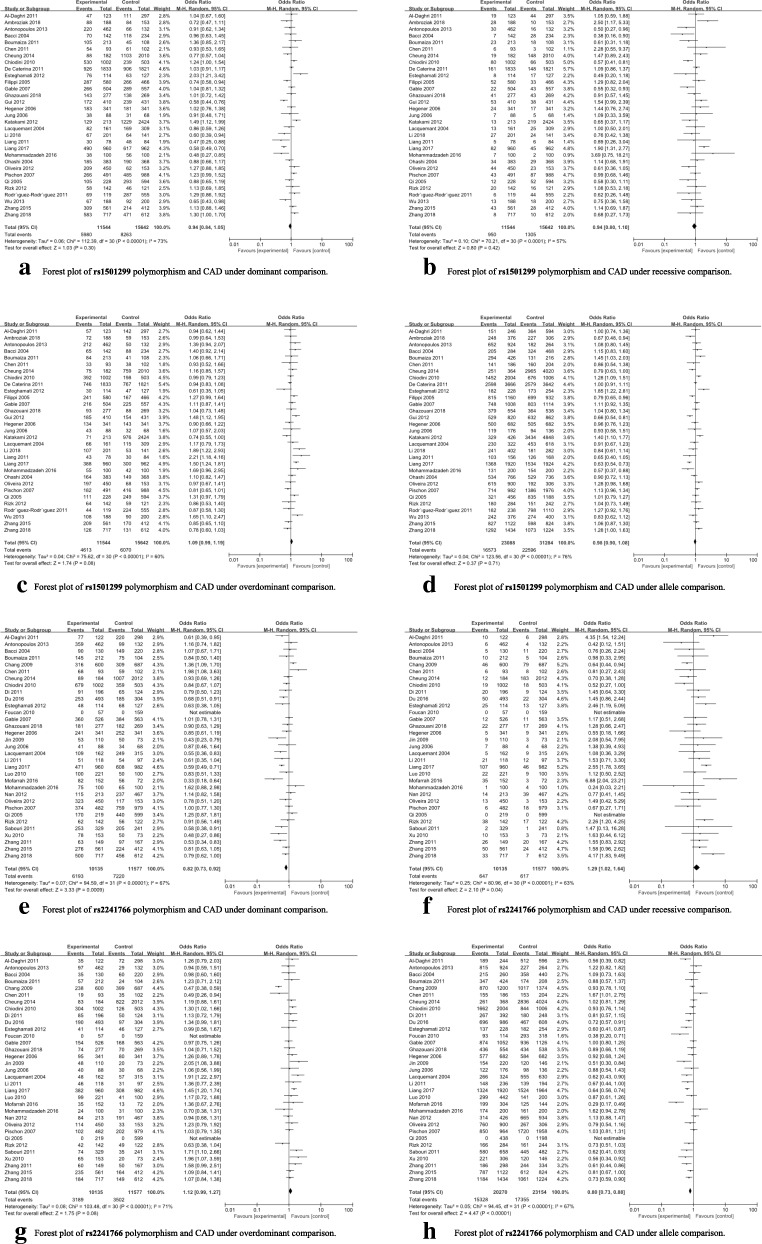
Forest plots for overall analyses of investigated polymorphisms. **a** Forest plot of rs1501299 polymorphism and CAD under dominant comparison; **b** Forest plot of rs1501299 polymorphism and CAD under recessive comparison; **c** Forest plot of rs1501299 polymorphism and CAD under overdominant comparison; **d** Forest plot of rs1501299 polymorphism and CAD under allele comparison; **e** Forest plot of rs2241766 polymorphism and CAD under dominant comparison; **f** Forest plot of rs2241766 polymorphism and CAD under recessive comparison; **g** Forest plot of rs2241766 polymorphism and CAD under overdominant comparison. **h** Forest plot of rs2241766 polymorphism and CAD under allele comparison

Results of overall and subgroup analyses were summarized in Table 2. To be brief, a significant association with the susceptibility to CAD was detected for rs2241766 (dominant model: *p* = 0.0009, OR = 0.82, 95%CI 0.73–0.92; recessive model: *p* = 0.04, OR = 1.29, 95%CI 1.02–1.64; allele model: *p* < 0.0001, OR = 0.80, 95%CI 0.73–0.88) polymorphism in overall analyses. Further subgroup analyses by ethnicity revealed that rs1501299 polymorphism was significantly associated with the susceptibility to CAD in East Asians, whereas rs2241766 polymorphism was significantly associated with the susceptibility to CAD in Caucasians, East Asians and South Asians. No any other positive results were observed in overall and subgroup analyses (see Table 2 and Fig. 2).

**Table 2:** Results of overall and subgroup analyses for *ADIPOQ* polymorphisms and CAD

Population	Sample size	Dominant comparison	Recessive comparison	Overdominant comparison	Allele comparison
		*P* value OR (95%CI) I^2^ statistic	*P* value OR (95%CI) I^2^ statistic	*P* value OR (95%CI) I^2^ statistic	*P* value OR (95%CI) I^2^ statistic
rs1501299 G/T		GG vs. GT + TT	TT vs. GG + GT	GT vs. GG + TT	G vs. T
Overall	11,544/15642	0.30 0.94 (0.84–1.05) 73%	0.42 0.94 (0.80–1.10) 57%	0.08 1.09 (0.99–1.19) 60%	0.71 0.98 (0.90–1.08) 76%
Caucasian	5481/5107	0.82 1.01 (0.93–1.09) 39%	0.12 0.80 (0.61–1.06) 67%	0.29 1.04 (0.96–1.13) 2%	0.47 1.04 (0.93–1.17) 64%
East Asian	4074/7814	0.08 0.82 (0.66–1.03) 82%	**0.03 1.20 (1.02–1.42)** 40%	0.10 1.18 (0.97–1.43) 76%	0.14 0.88 (0.74–1.04) 80%
South Asian	479/645	0.88 1.04 (0.61–1.77) 78%	0.97 0.99 (0.68–1.45) 42%	0.79 0.95 (0.65–1.38) 55%	0.90 1.03 (0.68–1.56) 80%
MI	4159/5883	0.67 1.04 (0.87–1.23) 65%	0.63 0.91 (0.63–1.32) 74%	0.42 0.96 (0.88–1.05) 47%	0.71 1.03 (0.88–1.21) 75%
rs2241766 T/G		TT vs. TG + GG	GG vs. TT + TG	TG vs. TT + GG	T vs. G
Overall	10,135/11577	**0.0009 0.82 (0.73–0.92)** 67%	**0.04 1.29 (1.02–1.64)** 63%	0.08 1.12 (0.99–1.27) 71%	**< 0.0001 0.80 (0.73–0.88)** 67%
Caucasian	2771/2106	0.09 0.89 (0.79–1.02) 27%	0.39 0.87 (0.62–1.20) 0%	**0.04 1.15 (1.01–1.32)** 33%	0.24 0.93 (0.84–1.05) 20%
East Asian	4856/6280	**0.02 0.80 (0.66–0.96)** 77%	0.06 1.35 (0.99–1.84) 68%	0.30 1.12 (0.90–1.40) 83%	**0.0006 0.80 (0.71–0.91)** 66%
South Asian	959/960	**0.04 0.69 (0.48–0.99)** 66%	**< 0.0001 2.67 (1.82–3.91)** 39%	0.76 1.05 (0.76–1.46) 56%	**0.01 0.64 (0.45–0.91)** 76%
MI	1869/1407	0.19 0.90 (0.77–1.05) 0%	0.11 0.68 (0.43–1.09) 18%	0.06 1.16 (0.99–1.36) 30%	0.48 0.95 (0.83–1.09) 0%

Abbreviations: *OR* Odds ratio, *CI* Confidence interval, *NA* Not available, *CAD* Coronary artery disease, *MI* Myocardial infarction

The values in bold represent there is statistically significant differences between cases and controls

### Sensitivity analyses

We performed sensitivity analyses by excluding studies that deviated from HWE. No alterations of results were detected in sensitivity analyses, which suggested that our findings were statistically reliable.

### Publication biases

Publication biases were evaluated with funnel plots. We did not find obvious asymmetry of funnel plots in any comparisons, which indicated that our findings were unlikely to be impacted by severe publication biases.

## Discussion

Based on combined analyses of 45 eligible studies, our study showed that rs1501299 and rs2241766 polymorphisms were both significantly associated with the susceptibility to CAD in certain populations, which suggested that these two polymorphisms may be used to identify individuals with higher susceptibility to CAD. There are two possible explanations for our positive findings. First, genetic variations of the *ADIPOQ* gene may lead to alternations in gene expression or changes in ADIPOQ protein structure, which may subsequently affect biological functions of ADIPOQ and ultimately impact individual susceptibility to CAD. Second, it is also possible that *ADIPOQ* polymorphisms may be linked to each other or even linked to other unidentified genes, which could also impact individual susceptibility to CAD.

There are several points that should be noted about this meta-analysis. Firstly, previous experimental studies demonstrated that mutant alleles of investigated polymorphisms could lead to decreased adiponectin generation, which may partially explain our positive findings [12–19]. Secondly, it is also worth noting that for rs1501299 polymorphism, the trends of associations in different ethnicities were not always consistent, and this may be attributed to ethnic differences in genotypic distributions of investigated polymorphisms. However, it is also that these inconsistent findings may be resulted from a complex interaction of both genetic and environmental factors. Thirdly, it should be noted that significant between-study heterogeneities were observed in all genetics comparisons of overall analyses, which may partially attributed to ethnic and racial differences of eligible studies. To overcome between-study heterogeneities, REMs were used for pooled analyses, and in further subgroup analyses, we noticed that between-study heterogeneities among studies that were conducted in Caucasians were relatively small, which also supported that ethnic background could impact individual susceptibility to CAD. Fourthly, a recent meta-analyses conducted by Hou et al. [24] also tried to explore potential associations between *ADIPOQ* polymorphisms and CAD. However, our findings should be considered as more conclusive compared to that of previous meta-analysis since many related studies were published in the last three years, which warranted an update meta-analysis. Totally 10 more eligible studies were enrolled in our pooled analyses, and the sample sizes of our analyses were also significantly larger than that of previous meta-analyses, which could significantly reduce the risk of obtaining false positive or false negative results. Compared with the previous meta-analysis, similar positive results were detected for rs2241766 polymorphism in overall and subgroup analyses. However, positive results in Caucasians for rs1501299 polymorphism were no longer observed in our meta-analysis. Instead, we found that rs1501299 polymorphism could impact individual susceptibility to CAD in East Asians under recessive genetic model. Therefore, future studies with larger sample sizes are still needed to test the potential associations between *ADIPOQ* polymorphisms and CAD, especially for rs1501299 polymorphism. Fifthly, our study only focused on two mostly investigated *ADIPOQ* polymorphisms, and future meta-analyses should try to investigate the associations between CAD and other common *ADIPOQ* polymorphisms such as rs266729, rs822395 and rs17300539. These polymorphisms were not analyzed by us because we failed to find any additional eligible studies compared to the previous meta-analysis conducted by Hou et al. [24].

Some limitations of this meta-analysis should also be acknowledged when interpreting our findings. First, our pooled analyses were based on unadjusted estimations due to lack of raw data, and failure to perform further adjusted analyses may impact the reliability of our findings [25, 26]. Second, since our pooled analyses were based on retrospective case-control studies, despite our positive findings, future perspective studies are still needed to examine whether there is direct causal relationship between *ADIPOQ* polymorphisms and CAD [27, 28]. Third, associations between *ADIPOQ* polymorphisms and CAD may also be modified by gene-gene and gene-environmental interactions. However, due to lack of raw data, we could not conduct relevant analyses [29, 30]. Fourth, our analyses were based on retrospective case-control studies. Thus, despite the relatively high NOS score, it was still possible that our findings might be impacted by potential selection, measurement and confounding biases. Taking the above mentioned limitations into consideration, our findings should be interpreted with caution.

## Conclusions

In conclusion, our meta-analysis suggested that rs1501299 and rs2241766 polymorphisms were both significantly associated with the susceptibility to CAD in certain populations. However, further well-designed studies are still warranted to confirm our findings.

## Abbreviations

*ADIPOQ*: Adiponectin
CAD: Coronary artery disease
HWE: Hardy-Weinberg equilibrium
NOS: Newcastle-Ottawa scale
